# Thalamostriatal System Controls the Acquisition, Performance, and Flexibility of Learning Behavior

**DOI:** 10.3389/fnsys.2021.729389

**Published:** 2021-10-18

**Authors:** Shigeki Kato, Kayo Nishizawa, Kazuto Kobayashi

**Affiliations:** Department of Molecular Genetics, Institute of Biomedical Sciences, Fukushima Medical University School of Medicine, Fukushima, Japan

**Keywords:** intralaminar thalamic nucleus, thalamostriatal pathway, pathway targeting, chemogenetic manipulation, learning, behavioral flexibility

## Abstract

The dorsal striatum (DS) is a key structure of the basal ganglia circuitry, which regulates various types of learning processes and flexible switching of behavior. Intralaminar thalamic nuclei (ILNs) provide the main source of thalamostriatal inputs to the DS and constitute multiple nuclear groups, each of which innervates specific subdivisions of the striatum. Although the anatomical and electrophysiological properties of thalamostriatal neurons have been previously characterized, the behavioral and physiological functions of these neurons remain unclarified. Two representative thalamostriatal cell groups in the parafascicular nucleus (PF) and the central lateral nucleus (CL) are located in the caudal and rostral regions of the ILNs in rodents. Recently, the behavioral roles of these thalamostriatal cell groups have been investigated by the use of genetic and pharmacological manipulation techniques. In the current review, we summarize behavioral studies on thalamostriatal neurons, showing the key roles of these neurons in different learning processes, such as the acquisition, performance, and flexibility of behavior.

## Introduction

The basal ganglia circuit plays important roles in controlling movement, motor learning, instrumental learning, and flexible switching of behavior (Graybiel, 2008; Belin et al., 2009; Floresco et al., 2009; Balleine and O’Doherty, 2010; Hikosaka et al., 2018). The dorsal striatum (DS) is the central structure that composes the basal ganglia circuit and regulates the output activity of the circuit through direct and indirect projection pathways (Alexander and Crutcher, 1990; Parent and Hazrati, 1995). Intralaminar thalamic nuclei (ILNs) provide the main source of thalamostriatal inputs to the DS and constitute multiple nuclear groups, each of which innervates specific subdivisions of the striatum (Groenewegen and Berendse, 1994; Van der Werf et al., 2002; Smith et al., 2004, 2014; Yasukawa et al., 2004; Mandelbaum et al., 2019). Thalamostriatal neurons make synaptic contacts on striatal projection neurons and cholinergic interneurons and elicit excitatory responses of these neurons (Lapper and Bolam, 1992; Ding et al., 2010). The two major nuclear groups in rodents are the parafascicular nucleus (PF) and the central lateral nucleus (CL), which are localized in the caudal and rostral regions of the ILNs, respectively. PF neurons form dense clusters of axonal varicosities containing many terminal boutons on striatal neurons, whereas CL neurons make sparsely axonal arborization with varicose collaterals on these neurons (Deschênes et al., 1996a,b; Unzai et al., 2017). The two types of ILN neurons have heterogeneous electrophysiological properties with differences in the functional characteristics of their striatal synapses and the expression pattern of ionotropic glutamate receptor subtypes (Lacey et al., 2007; Ellender et al., 2013).

As compared to the anatomical and physiological evidence for properties of ILN neurons, studies on the behavioral and physiological roles of these neurons have been limited. Early studies with the excitotoxic lesion of the ILNs in rodents have reported the involvement of these neurons in several types of memory and learning paradigms (Burk and Mair, 1998; Massanés-Rotger et al., 1998; Mair et al., 2002). Recently, we have developed a novel technology for pathway-specific manipulation with a lentiviral vector showing highly efficient retrograde gene transfer (HiRet) to investigate the behavioral and physiological functions of neurons of interest (see for reviews, Kobayashi et al., 2018; Kato and Kobayashi, 2020). We applied this technology to study the roles of the thalamostriatal system derived from the PF and CL in basal ganglia circuit functions (Kato et al., 2011a, 2018). In addition, by combining genetic and pharmacological manipulation techniques, the roles of the ILN-DS circuit have been investigated for various types of learning behavior (Saund et al., 2017; Li et al., 2018; Melief et al., 2018; Xiao et al., 2018; Cover et al., 2019; Johnson et al., 2020). In the current review, we summarize behavioral studies on thalamostriatal neurons, showing the key roles of these neurons in different learning processes, such as the acquisition, performance, and flexible switching of behavior.

## Role of PF Thalamostriatal System in Learning Processes

**Figure 1:**
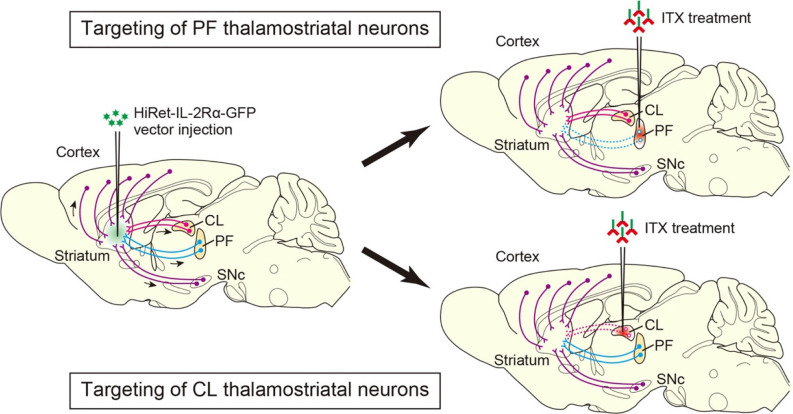
Selective neural pathway targeting of thalamostriatal neurons arising from the PF and CL. The highly efficient retrograde gene transfer (HiRet)-IL-2Rα-GFP vector is injected into the dorsal striatum (DS) and transported retrogradely through axons to various brain regions, in which the expression of the IL-2Rα-GFP transgene is induced. ITX is then treated into the PF and CL, resulting in the elimination of thalamostriatal neurons expressing the transgene in the PF and CL, respectively. Arrows show retrograde axonal transport of the viral vector. Dotted lines indicate the removal of the corresponding neural pathways. HiRet, highly efficient retrograde gene transfer; IL-2Rα, interleukin-2 receptor α-subunit; GFP, green fluorescent protein; ITX, immunotoxin; PF, parafascicular nucleus; CL, central lateral nucleus.

To address the behavioral functions of PF neurons projecting to the DS, we performed selective neural pathway targeting, which eliminates the thalamostriatal pathway derived from the PF in mice (Kato et al., 2011a). The HiRet vector, which is a pseudotype of human immunodeficiency virus type-1 lentiviral vector with a fusion glycoprotein composed of rabies virus and vesicular stomatitis virus glycoprotein segments, enhances retrograde gene transfer into neurons (Kato et al., 2011b; Kobayashi et al., 2018; Kato and Kobayashi, 2020). HiRet vector encoding human interleukin-2 receptor α-subunit (IL-2Rα), fused to an enhanced green fluorescent protein (IL-2Rα-GFP) was injected into the DS, and then a recombinant immunotoxin (ITX) was treated into the PF (see Figure 1). This treatment efficiently removed the PF-derived thalamostriatal neurons, and the cell number was significantly reduced as compared to the control group, which received the injection of phosphate-buffered saline (PBS) into the PF (approximately 30% of the control cell number), together with a great decrease of the nerve terminals innervating the striatum in the ITX-treated group. These anatomical data confirmed the selective, efficient elimination of PF-derived thalamostriatal neurons in the HiRet-IL-2Rα-GFP vector-injected mice after ITX treatment.

Mice lacking PF-derived thalamostriatal neurons were subjected to some behavioral tasks that are considered to require the function of the DS (Kato et al., 2011a). Spontaneous locomotor activity and drug (methamphetamine)-induced motor activation were normal in the ITX-treated mice, and motor skill learning monitored using the rotarod test was unaltered in these animals. The mice were also found to have a normal reaction time in a simple lever press task. Next, the mice were tested for a two-choice reaction time task dependent on visual stimulus (visual discrimination task). The mice lacking PF thalamostriatal neurons showed a reduction in the correct response ratio and lengthening in the correct response time as compared to the control mice, indicating impairment in the acquisition of discrimination learning. When ITX treatment was carried out after the task acquisition, the mice displayed a dramatic decline in the correct response ratio compared to the control animals with no change in the correct response time. These data suggest that PF thalamostriatal neurons play key roles in the acquisition and performance of the visual discrimination task, although their roles in reaction time control in the performance phase appear to be transferred to other brain regions. The results of the behavioral analysis for PF thalamostriatal neurons are summarized in Table 1.

**Table 1:** Behavioral impacts of selective targeting of PF and CL thalamostriatal neurons.

Behavioral paradigm	PF	CL
Locomotor activity	Spontaneous: normal	Spontaneous: normal
	Drug-induced: normal	Drug-induced: normal
Motor skill learning	Normal	Normal
Simple lever press	Normal	Normal
Visual discrimination	Acquisition: reduced correct response ratio, lengthened correct response time	Acquisition: normal correct response ratio, normal correct response time
	Performance: reduced correct response ratio, normal correct response time	Performance: transiently reduced correct response ratio, lengthened correct response time
Reversal learning	Not tested	Impaired performance
Attentional set-shifting	Not tested	Impaired performance

*Behavioral data for PF and CL neurons are from references by Kato et al. (2011a) and Kato et al. (2018), respectively. PF, parafascicular nucleus; CL, central lateral nucleus*.

## Role of CL Thalamostriatal System in Learning Processes

To investigate the behavioral functions of CL neurons innervating the DS, selective neural pathway targeting technology was used to remove the thalamostriatal pathway originating from the CL in mice (Kato et al., 2018). After the injection of the HiRet-IL-2Rα-GFP vector into the DS, ITX treatment was carried out into the CL (see Figure 1). This treatment resulted in the ablation of CL-derived thalamostriatal neurons (approximately 47% of the control cell number). The optical stimulation of CL neurons expressing channelrhodopsin-2 evoked local field potentials in the DS in the control group, whereas the same treatment generated a much smaller peak amplitude of the evoked potentials in the ITX-treated group. These anatomical and electrophysiological results ascertained the selective, efficient elimination of CL-derived thalamostriatal neurons in the vector-injected mice that received ITX treatment.

Mice lacking CL-derived thalamostriatal neurons were used for the behavioral tasks involved in striatal functions (Kato et al., 2018). Spontaneous locomotor activity, drug-induced motor activation, motor skill learning, and simple lever press were normal in the ITX-treated mice. The mice were then tested for the visual discrimination task and showed no changes in the correct response ratio and correct response time in the acquisition phase of the task. However, in the performance phase, they indicated the transient reduction of correct response ratio that was gradually recovered to the normal level during the daily repetition of the trials. Interestingly, the correct response time continued to lengthen compared to the control level, not showing the recovery during the task period. These data suggest that CL thalamostriatal neurons play important roles in the performance but not the acquisition of visual discrimination, although their roles in correct response were temporal. The functions to control the correct response time may be transferred from the PF neurons to CL neurons during the progress of discrimination learning. The results of behavioral analysis for CL thalamostriatal neurons are summarized in Table 1.

Furthermore, a chemogenetic suppression was executed for CL thalamostriatal neurons by using the designer receptors exclusively activated by designer drugs (DREADD) system with an inhibitory human M4 muscarinic receptor coupled to Gi (hM4Di; Kato et al., 2018). There was no effect of a selective agonist for the receptor clozapine-N-oxide (CNO) treatment on the performance of the visual discrimination task. The discrepancy in the behavioral effects of genetic manipulation between the pathway targeting and chemogenetic manipulation suggests the presence of some compensatory mechanisms to restore changed neuronal activity against the chemogenetic manipulation. Although the detailed mechanisms that explain the compensation are unclear, there may be some compensation mechanisms in response to chronic exposure of the agonists, such as the desensitization (Gupta and Mishra, 1993; Mullaney et al., 1993), internalization (Claing et al., 2002; Gainetdinov et al., 2004; Kelly et al., 2008), and down-regulation of the receptors or coupled G proteins (Karoor et al., 1996; Tsao and von Zastrow, 2000). Indeed, CNO-induced chronic inhibitory effects are reported to cause compensatory adaptation (Soumier and Sibille, 2014; Carvalho Poyraz et al., 2016). Another possibility to explain the compensation of thalamostriatal neurons is that the agonist-induced suppression is partial and the remaining activity of neurons is sufficient for the correct execution of visual discrimination.

## Role of CL Thalamostriatal System in Behavioral Flexibility

The CL is reported to innervate the boundary of the dorsomedial striatum (DMS) and dorsolateral striatum (Yasukawa et al., 2004). The DMS is required for flexible switching of motor responses (Castañé et al., 2010; Braun and Hauber, 2011), and the DMS receives massive projections from the medial prefrontal cortex (Gabbott et al., 2005). These findings suggest the involvement of CL thalamostriatal neurons in flexible switching of behavior. We thus tested the roles of CL thalamostriatal neurons in behavioral flexibility by using mice lacking these neurons (Kato et al., 2018). First, the reversal of place discrimination was tested, in which mice acquire the correct lever based on place discrimination, and then the position of the reinforced lever was changed to the opposite side. The mice lacking CL thalamostriatal neurons learned normally in the acquisition phase, but they had impairment in the reversal learning, showing a significant increase in the session number to reach the criterion (>90% of three consecutive session average) as compared to the control group. Next, attentional set-shifting was tested, in which the strategy for the visual discrimination is converted to the place discrimination strategy. This behavioral task evaluates the ability for cognitive function that adapts behavior to the changed strategy and cancels the previous strategy. The mice acquired normally the visual discrimination, but they showed impaired attentional set-shifting, revealing a larger session number to reach the criterion (>90% of three consecutive session average) as compared to the control group. These data suggest that CL thalamostriatal neurons are required for flexible switching of motor actions in response to changes in choice or strategy. The behavioral analysis results for CL thalamostriatal neurons are summarized in Table 1.

Chemogenetic suppression by the DREADD system was carried out to confirm the role of CL thalamostriatal neurons in behavioral flexibility (Kato et al., 2018). The agonist treatment impaired both the performance of reversal learning and attentional set-shifting in mice expressing hM4Di receptor in CL neurons. These data support the findings obtained from the selective pathway targeting of CL thalamostriatal neurons, confirming a facilitative role of these neurons in flexible switching of behavior. A chemogenetic experiment was effective on reversal learning and set-shifting paradigms, but not on the performance of visual discrimination. These results suggest a difference in the susceptibility of specific behaviors to chemogenetic manipulation. Discriminative behavior may be normalized by some compensatory mechanisms for receptor function responses and signaling pathways against repetitive exposure of the agonist or by remaining neuronal activity due to partial inhibition of the receptor signaling, as described in the previous section.

## Contribution of ILN Neurons to Other Learning Behaviors

Some recent studies with genetic and pharmacological manipulations of ILN neurons provide useful information on the roles of thalamostriatal pathways in various types of learning behavior. Chemogenetic inhibition of PF neurons projecting to the DMS using the DREADD system had no effect on the accuracy and latency of correct responses, but increased the probability of perseverative errors in a five-choice reaction time task, suggesting that the PF-DMS pathway plays a critical role in behavioral flexibility and response inhibition (Saund et al., 2017). This report, together with our analysis of the CL-DS pathway, supports the importance of the thalamostriatal pathway in behavioral flexibility, although the thalamostriatal subgroups and behavioral paradigms used are different between the studies. In contrast, chemogenetic inhibition of ILN neurons innervating the DS impaired alternation response in a T-maze task, but it did not influence the reversal learning in the maze (Xiao et al., 2018). Conditional knockout of the gene encoding vesicular glutamate transporter 2 in ILN neurons, leading to reduced glutamatergic transmission in the DS, showed impaired motor coordination and slower responses in water maze and two-way active avoidance tasks, but there were no effects on reversal learning and set-shifting with a water maze task (Melief et al., 2018). The results of behavioral flexibility function in these reports are contradictory to the data of the aforementioned two studies, and the contradiction may be because of the differences in the experimental strategies, ranges of target areas, and behavioral tasks used.

In addition, optical stimulation of ILN-derived glutamatergic terminals in the DMS served as a reinforcer in a self-paced operant task, and evoked dopamine release and excitatory postsynaptic currents in striatal neurons in the DMS (Johnson et al., 2020). Optogenetic activation of the rostral ILN terminals in the DS mimicked operant responding that had been previously reinforced by food, which was blocked by dopamine D1 receptor antagonist (Cover et al., 2019). Pharmacological treatments with GABA receptor agonists to the anterior ILNs and with dopamine D1 receptor antagonist to the DMS also decreased methamphetamine seeking following forced abstinence (Li et al., 2018). These results indicate that the thalamostriatal pathway mediates reinforcement dependent on dopamine transmission.

The behavioral functions of ILN neurons in non-human primates were investigated by using single-unit recording and pharmacological inhibition of neuronal activity (see for a review, Kimura et al., 2004). The center médian (CM) and PF neurons in macaque monkeys exhibited responses to a novel sensory stimulus (visual, auditory, or somatosensory stimulus) and these neuronal responses gradually decreased to repeated exposure of the same stimulus (Matsumoto et al., 2001). Inactivation of CM/PF neurons by local infusion of muscimol abolished neuronal responses in the striatum to the stimulus associated with reward, suggesting that the CM/PF activity is correlated to the attentional process involved in the detection of an unpredictable stimulus (Matsumoto et al., 2001). Monkeys were trained to gaze a visual cue presented at the same location (validly cued target), and they exhibited a shorter reaction time to the validly cued target than to another visual cue presented on the opposite side (invalidly cued target; Minamimoto and Kimura, 2002). Pharmacological inhibition of CM/PF neurons resulted in the lengthening of the reaction time to the validly cued target without any effect on the responses to the invalidly cued target, supporting a key role of these neurons in the process of attention orienting to sensory events (Minamimoto and Kimura, 2002). In addition, CM neurons were activated in the situation when animals were forced to perform a small-reward action, and the activation was greater when a large-reward option was expected (Minamimoto et al., 2005). Electrical stimulation of the CM after the large reward action request caused a lower task performance, suggesting the contribution of CM neurons to a mechanism complementary to the response bias to the large reward (Minamimoto et al., 2005). The detailed analysis of these thalamostriatal functions in the primate brains is expected to be examined by using selective manipulation technology in the future.

## Future Aspects

We described the behavioral roles of PF and CL thalamostriatal neurons on the basis of the findings obtained from selective neural pathway targeting and chemogenetic manipulation. These data show that the two representative thalamostriatal neurons have distinct roles in the acquisition and performance phases of stimulus-dependent discrimination learning and that CL neurons are essential for the flexible switching of behavior in response to changes in choices or strategies. In addition, we summarized behavioral studies on thalamostriatal neurons obtained from other studies with genetic and pharmacological manipulation techniques. These studies demonstrate the important roles of thalamostriatal neurons in various types of learning behavior, such as motor skills and reinforcement learning. In the non-human primate brains, thalamostriatal neurons appear to be involved in an attentional process to sensory events, and a complementary process of decision and action bias.

The next important issue is how PF and CL thalamostriatal neurons regulate the learning processes and behavioral flexibility through the complex neural circuit. First, we need to determine the inputs to thalamostriatal neurons that are responsible for behavior. The ILNs receive dense innervations from various brain regions, such as the cerebral cortex, globus pallidus, superior colliculus, and cerebellum (Angaut et al., 1985; Krout et al., 2001; Van der Werf et al., 2002; Mastro et al., 2014). For instance, selective neural pathway targeting of the dentate nucleus in the cerebellum projecting to the CL indicates that cerebellar input plays an important role in motor skills in the rotarod and reaching tasks with no effect on spatial recognition and its behavioral flexibility (Sakayori et al., 2019). A recent study reports that a subgroup of globus pallidal neurons appears to regulate locomotion and reversal learning through projections to the PF (Lilascharoen et al., 2021). Inputs from other brain regions to the ILNs are expected to be defined for their functions in the behavior.

Second, it is necessary to understand that the mechanism underlying thalamostriatal inputs modulates striatal neuronal activity to control the behavior. The ILN neurons directly innervate cholinergic interneurons in addition to medium spiny neurons in the striatum, showing their intrinsic electrophysiological properties and functional characteristics on striatal synapses (Lapper and Bolam, 1992; Lacey et al., 2007; Ellender et al., 2013; Doig et al., 2014). In a brain slice preparation, optical stimulation of PF thalamostriatal axons generated patterned firing activity of cholinergic interneurons, which appeared to presynaptically gate corticostriatal inputs (Ding et al., 2010). Thalamostriatal neurons also regulate the activity of indirect pathway neurons depending on cholinergic interneurons through presynaptic control of thalamostriatal terminals (Tanimura et al., 2019). The stimulation of thalamostriatal terminals, through cholinergic interneuronal activation, triggers dopamine release in the DS (Threlfell et al., 2012; Cover et al., 2019). These data suggest that several types of learning behavior are mediated by the interaction of acetylcholine and dopamine transmission. In addition, striatal acetylcholine has also been reported to facilitate behavioral flexibility (Ragozzino et al., 2002; Tzavos et al., 2004), and thalamostriatal inputs to cholinergic interneurons appear to modulate goal-directed behavior after changes in action-outcome contingency (Bradfield et al., 2013). In contrast, selective targeting of striatal cholinergic interneurons reveals an inhibitory role of these neurons in reversal learning of place discrimination (Okada et al., 2014), and this effect is dependent on the trial spacing and discrimination type constituting the learning tasks (Okada et al., 2018). The neural substrates on how the thalamostriatal system, through striatal cholinergic interneurons, organizes the activity of the basal ganglia circuit and the resultant switching of behavior in response to changed contingency remain to be investigated in the future.

Finally, postmortem studies of Parkinson’s disease patients have reported degeneration of ILNs in addition to nigrostriatal dopamine neurons (Henderson et al., 2000; Lanciego et al., 2009; see for a review, Smith et al., 2014). The numbers of ILN neurons and their synaptic terminals in the DS are also reduced in parkinsonian monkeys treated with 1-methyl-4-phenyl-1,2,3,6-tetrahydropyridine (Villalba et al., 2014, 2019). Dopamine depletion increases N-methyl-D-aspartate (NMDA) receptor-mediated currents onto indirect striatal spiny neurons *via* the corticostriatal pathway (Warre et al., 2011) and suppresses NMDA currents onto direct striatal spiny neurons *via* the thalamostriatal pathway (Parker et al., 2016), which induces imbalance of these inputs and contributes to Parkinson’s disease. In addition, dopamine depletion leads to depotentiation of NMDA currents in PF synapses onto cholinergic interneurons, suggesting that the changed glutamatergic transmission may partially explain the pathology of the disease (Aceves Buendia et al., 2019). However, the effect of ILN degeneration on Parkinson’s disease symptoms has not yet been defined. The behavioral data obtained from selective neural pathway targeting suggest that the loss of ILN neurons may be associated with some cognitive deficits in Parkinson’s disease, related to the appropriate selection and flexible switching of behavior. Further study is needed to elucidate the impact of ILN degeneration on the disease state.

## Author Contributions

SK: writing—original draft, review and editing, drawing illustrations. KN: writing—review and editing. KK: writing—original draft, review and editing. All authors contributed to the article and approved the submitted version.

## Conflict of Interest

The authors declare that the research was conducted in the absence of any commercial or financial relationships that could be construed as a potential conflict of interest.

## Publisher’s Note

All claims expressed in this article are solely those of the authors and do not necessarily represent those of their affiliated organizations, or those of the publisher, the editors and the reviewers. Any product that may be evaluated in this article, or claim that may be made by its manufacturer, is not guaranteed or endorsed by the publisher.

## Abbreviations

CL: central lateral nucleus
DS: dorsal striatum
DMS: dorsomedial striatum
DREADD: designer receptors exclusively activated by designer drugs
GFP: green fluorescent protein
HiRet: highly efficient retrograde gene transfer
IL-2Rα: interleukin-2 receptor α-subunit
ILN: intralaminar thalamic nucleus
hM4Di: inhibitory human M4 muscarinic receptor coupled to Gi
ITX: immunotoxin
NMDA: N-methyl-D-aspartate
PBS: phosphate-buffered saline
PF: parafascicular nucleus.
